# Setting the rules of engagement: a qualitative study of challenges and enablers of engaged research in a South African emergency care system

**DOI:** 10.1186/s40900-026-00885-6

**Published:** 2026-04-15

**Authors:** Robert Holliman, Willem Stassen, Colleen Saunders

**Affiliations:** University of Cape Town, Cape Town, South Africa

**Keywords:** Patient and public involvement, Engaged research, Barrier and facilitators

## Abstract

**Background:**

Engaged research requires collaboration with community stakeholders across the research process, yet little is known about its practice in low- and middle-income countries. The Western Cape in South Africa, which includes Cape Town as its largest city, combines substantial socio-economic inequality with strong emergency care academic capacity, offering a distinctive environment in which to explore these dynamics. This study aimed to examine stakeholders’ perspectives on the barriers to and enablers of practising engaged research.

**Method:**

This qualitative study used semi-structured interviews to explore experiences of engaged research among a broad spectrum of stakeholders. Purposive and snowball sampling ensured wide representation across public, clinical, academic, managerial, and policy groups. Data were analysed using inductive content analysis. Reporting was guided by Consolidated Criteria for Reporting Qualitative Research (COREQ) and Guidance for Reporting Involvement of Patients and Public v2 (GRIPP2) to ensure trustworthiness.

**Results:**

Thirty interviews were completed, with participants offering insights informed by a range of experiences, including overlapping roles across the emergency care community. Analysis generated four overarching categories describing the factors shaping engaged research in this setting: (1) Cultivating relationships and spaces for engagement; (2) Overstretched and under-resourced; (3) Systems, structures, and scholarship; and (4) The rules of engagement. These categories highlighted both key barriers and opportunities for strengthening engagement practices.

**Conclusion:**

Meaningful engaged research depends on sustained relationships and shared accountability between academic and non-academic partners. Although stakeholders viewed engagement as necessary and achievable, it is constrained by limited available time, fragmented approval systems, uncertainty about how to participate, and low trust. Strengthening engagement requires early relationship building, structured opportunities to develop engagement skills, and better alignment of degree research with ongoing community priorities. Establishing transparent and mutually agreed rules of engagement is central to embedding collaboration and sustaining trust over time.

**Plain English summary:**

Research is more useful when the people affected by it help shape it. This process is often termed engaged research. Although it is becoming more common, we still know little about how to carry out engaged research in low resource settings. This study took place in the Western Cape province of South Africa. The region has major social inequality but also a developing emergency care system. This mix makes it a good place to explore what helps and what gets in the way of engaged research in emergency care. We spoke to thirty people from different backgrounds, including the public, healthcare workers, managers, policymakers, and researchers. They shared their experiences of trying to work together as partners on research projects. We found several points, summarised under four main messages. First, building relationships is essential. People need time and supportive spaces to talk, understand each other, and build trust. Second, many groups are overstretched. Busy services, limited time, and few resources make it hard to stay involved. Third, systems and structures, including approval processes, can slow or block progress but could improve with better support. Finally, people wanted clearer rules of engagement so everyone knows their roles, responsibilities, and how decisions will be made. Overall, engaged research is valued and possible but needs support. Participants highlighted the importance of time for relationship building, addressing barriers within approval processes, and improving training and guidance for community members, health care workers, and researchers. These improvements can lead to stronger partnerships, better research, and benefits for communities.

**Supplementary Information:**

The online version contains supplementary material available at 10.1186/s40900-026-00885-6.

## Background

Broadly defined, engaged research refers to the collaborative generation of knowledge through meaningful partnerships between academic and non-academic stakeholders [1, 2]. Conceptually similar with community-based participatory research [3], integrated knowledge translation [4], and partnership research [5], the scholarship emphasises community collaboration throughout the research process, from shaping research questions to disseminating findings [2]. These approaches, along with terms such as community-engaged research and engaged scholarship, are often used interchangeably with engaged research to describe a shared emphasis on collaborative knowledge production between academic and non-academic stakeholders. The concept of community itself is contested, with extensive scholarship highlighting its breadth and ambiguity [6–8]. In previous work, we examined how the emergency care (EC) community can be defined within a health system context, describing it as a broad and variable network of stakeholders connected through shared practice, governance, or lived experience [9]. Increasingly, engaged research is seen as a way to enhance the relevance, uptake, and impact of academic work, requiring early and continuous involvement of community members such as policy-makers, practitioners, and the public [2].

The motivation for engaged research is well established, with evidence showing that involving non-academic partners increases the likelihood that findings are implemented in practice and policy [2, 10, 11] and that decision-makers are more likely to act on evidence they have helped generate [12]. Furthermore, these collaborations have been associated with capacity building among participants, including the development of leadership and workforce skills, and enhanced institutional capacity [13]. These benefits are particularly important in healthcare, where persistent gaps between evidence generation and translation into practice continue to hamper service delivery [14].

Engaged research is well suited to EC, where rapid, often life-saving responses place EC at a critical interface between health systems and the communities they serve. Contextually grounded, engaged research is more likely to generate actionable and locally relevant outcomes, particularly in low- and middle-income countries (LMICs) where the burden of acute illness and injury is disproportionately high and effective EC can significantly reduce deaths and disability [15, 16].

South Africa faces many of the structural inequities and resource constraints common to other LMICs, and its EC system remains in development despite substantial progress over the past two decades [17]. Within South Africa, the Western Cape province, hosts one of Africa’s most established academic EC communities, supported by a long-standing training programme and strong research output [18, 19]. At the same time, the Western Cape experiences significant inequality and widespread poverty that place sustained pressure on its health system [20]. This combination of recognised academic strength and persistent socio-economic challenges makes the Western Cape a particularly valuable setting in which to explore engaged research, as it illustrates both the opportunities for meaningful impact and the practical challenges that shape its implementation.

Despite growing recognition of its value, evidence on how engaged research operates in practice remains limited, particularly in LMICs. While high-income countries (HIC) have invested in collaborative centres and piloted various models [21, 22], there is still no widely accepted framework for understanding or evaluating engaged research [14, 23]. Reviews consistently point to limited robust studies on the practice of engaged research, observing that existing literature concentrates on narrow elements, notably policy uptake, instead of broader relationships or stages such as co-design and implementation [4, 23–25]. Consequently, understanding of the processes and conditions that enable engaged research, especially in LMICs, remains limited.

This study draws on the concept of patient and public involvement (PPI), defined as research being carried out ‘with’ or ‘by’ members of the public rather than ‘to’, ‘about’ or ‘for’ them [26]. Within this study, PPI is understood as one approach within the broader paradigm of engaged research, representing a well-established tradition that emphasises the active involvement of patients and communities in the research process. This aligns with broader understandings of engaged scholarship as an active partnership between stakeholders and researchers in the production of new healthcare knowledge and evidence [27]. Although PPI is well established in HIC, studies in LMICs emphasise the need to adapt PPI principles to local systems, power structures, and community realities [28, 29]. In this paper, we therefore adopt engaged research as the primary analytical lens, using it consistently to describe collaborative approaches to knowledge generation. This study therefore responds to a growing number of calls to promote more engaged forms of scholarship that involve communities throughout the research process to help close persistent knowledge–practice gaps [5, 30, 31]. This is especially critical in LMICs where strengthening local ownership and contextual relevance of research are key to sustainable health system improvement. This study aimed to explore the perspectives of Western Cape EC stakeholders on the barriers to, and enablers of, practising engaged research in this setting.

## Methods

### Study setting

The Western Cape is one of South Africa’s nine provinces and has a population of over seven million people [32]. The province recorded an unemployment rate of 20.4% in 2023 [32]. Recent estimates indicate that up to 18.5% of households are at risk of running out of money for food, alongside persistently high rates of violence and injury that increase demand for emergency services [33]. Together, these indicators reflect sustained socio-economic pressures. Most residents speak one of three commonly used languages, namely English, isiXhosa, or Afrikaans, although South Africa has eleven official languages. The Western Cape includes both densely populated urban centres including Cape Town as its largest city, and geographically dispersed rural districts, where differences in infrastructure, workforce distribution, and service availability contribute to disparities in healthcare access and outcomes. EC in the Western Cape is delivered through both public and private sectors, with roughly three-quarters of the population reliant on public services [32] via an integrated network of hospitals, clinics, and emergency medical services, spanning out-of-hospital and facility-based care [32].

### Author characteristics and credentials

RH is a qualified emergency medicine specialist who has been engaged in the Western Cape EC community since 2020. He holds an MBBS, a BSc and is currently pursuing a PhD in emergency medicine. WS is an associate professor with a background in emergency medicine and paramedicine, convenes a Western Cape-based PhD programme in emergency medicine, holds a PhD, and has extensive qualitative research experience. Both RH and WS identify as men. CS is a woman, holds a PhD, and is also an associate professor at a leading Western Cape academic institution.

### Study population and design

This study is part of a wider examination of engaged research in the Western Cape EC community, informed by earlier work defining the community [9] and its stakeholder relationships. It was grounded in a relativist, constructivist worldview, which assumes that knowledge is co-produced through social interaction. Semi-structured interviews were used to capture participants’ perspectives, allowing flexibility in exploring individual experiences while maintaining consistency across key topics. The Consolidated Criteria for Reporting Qualitative Research (COREQ) [34] and Guidance for Reporting Involvement of Patients and the Public version 2 (GRIPP2) [35] checklists were used to guide reporting.

A two-stage sampling approach was used to capture a broad range of perspectives from the Western Cape EC community. In the first stage, purposive sampling guided initial recruitment across diverse stakeholder groups, informed by the seven Ps stakeholder taxonomy (Patients, Public, Providers, Purchasers, Payers, Policymakers, Product Makers, and Principal Investigators), which was used as a guiding framework to ensure broad representation across relevant stakeholder domains [36]. In the second stage, snowball sampling was used, with participants asked to identify others who held relevant insights or played important roles in the EC system.

Data were collected through interviews conducted via an online platform (Microsoft Teams, Microsoft Corporation, Redmond, Washington, U.S.). Online interviews were selected to facilitate participation across geographically dispersed districts and to accommodate participants’ clinical and administrative schedules. Although face-to-face methods may support rapport, the virtual format reduced logistical barriers and enabled broader access to stakeholders. Interviews explored participants’ experiences with engaged research in the Western Cape EC community, the barriers limiting its practice, and current and potential enablers of wider adoption. A discussion guide was developed from a review of engaged research literature and refined throughout the study as new insights emerged (Appendix A). All interviews were conducted by RH, with early sessions observed by CS or WS, who provided formative feedback. Interviews were not blinded, as participants were aware of the interviewer’s identity; however, confidentiality was maintained through anonymisation in transcription and reporting. Two preliminary interviews with CS and WS were used to pilot the process, and the resulting data were included in the final analysis. All interviews were conducted in English, audio recorded for accuracy, and transcribed verbatim by RH. Transcripts were reviewed alongside the recordings to ensure accuracy, and selected transcripts were discussed with CS and WS during analysis to support credibility. Audio files and anonymised transcripts were stored on a password-protected laptop and a secure institutional online storage system accessible only to the named investigators. Field notes were taken to complement the transcripts and inform interpretation.

### Data analysis

Data were analysed using inductive-dominant content analysis at the manifest level. NVivo Pro (v.14; QSR International, Burlington, Massachusetts, U.S.) supported coding and data organisation. The analytic process was structured using key procedural elements of Braun and Clarke’s framework, including familiarisation with the data, initial coding, categorisation, reviewing categories, and defining and naming categories, to support a systematic and transparent approach to analysis [37]. An initial coding framework was developed early in the analysis and refined as data collection progressed in response to emerging ideas and patterns. A hybrid inductive-deductive strategy was then used for the remaining transcripts, with new codes added when needed and existing codes applied systematically to ensure consistency. RH led the primary coding, while WS and CS contributed to code refinement through collaborative discussion and iterative review. Participants were provided with the opportunity to review their transcripts and suggest corrections or clarifications if desired. This was offered individually, and no participants requested any amendments.

### Trustworthiness

To ensure qualitative rigour, this study was guided by Guba’s framework of credibility, transferability, dependability, and confirmability [38]. Credibility was supported through the use of established methods and existing literature to inform the interview schedule, coding framework, and analytical approach. Written informed consent was obtained from all participants prior to interview, including explicit consent for audio recording. Consent was documented electronically in advance of participation. Interviews were conducted using an institutionally licensed online platform, and recording commenced only after participants had reconfirmed their agreement. The interview process was iterative, with the guide refined through pilot interviews, post-interview reflection, and team debriefs [39]. Transferability is supported by a clear description of the study setting, allowing readers to consider contextual factors such as language, employment, and other socio-demographic characteristics that may influence engagement practices. Dependability was strengthened through transparent documentation of data collection and analytical procedures, facilitating potential replication [38, 39]. Confirmability was enhanced through regular team discussions and critical reflection, with the COREQ and GRIPP2 checklists used to support transparency across the reporting process and reinforce both confirmability and dependability [34, 35].

## Results

A total of 30 interviews were completed. Nineteen EC stakeholders initially agreed to participate, and a further 11 were identified through snowball sampling. All individuals who responded consented to take part, and none withdrew, although five individuals did not respond to the initial invitation. The sample reflected diverse perspectives across the EC system, including out-of hospital providers (prehospital EC personnel such as ambulance service staff), EC clinicians, academics, managers, policy-makers, patient advocates (e.g. individuals representing patient perspectives within health-related forums or health care site facilitator), community representatives (e.g. individuals involved in local councils, community initiatives, or university-linked projects), and degree candidates enrolled in masters or doctoral programmes (Table 1). These groupings reflect context-specific roles identified within the Western Cape EC system, informed by, but not limited to the stakeholder domains outlined in the sampling framework. Clinical background refers to participants’ primary professional training or area of practice, recognising that many held overlapping roles across clinical, academic, managerial, and community domains. The “Current role” reflects the participant’s primary or self-identified role at the time of interview, acknowledging that multiple roles may have been held concurrently. Participants are listed numerically to preserve anonymity rather than grouped by role or sector. Eighteen participants self-identified as women and 12 as men. Interviews lasted 36 to 66 min, with an average duration of 51 min, and no repeat interviews were undertaken.

**Table 1:** Demographic and professional characteristics of interview participants

Participant Number	Gender	Clinical Background	Current Role	Sector
P1	Male	Out-of hospital provider	Researcher	Public
P2	Female	None	Researcher	Public
P3	Male	Emergency Physician	Joint staff: Clinical & Academic	Public
P4	Female	Emergency Physician	Joint staff: Clinical & Academic	Public
P5	Male	Emergency Physician	Joint staff: Clinical & Academic	Public
P6	Female	Nurse	Researcher	Public
P7	Female	Emergency Physician	Joint staff: Clinical & Academic	Public
P8	Male	Physician	Researcher	Public
P9	Male	Emergency Physician	Academic	Public
P10	Female	Emergency Physician	Governance	Public
P11	Female	Public Health	Governance	Public
P12	Female	Nurse	Joint staff: Clinical & Academic	Public
P13	Female	Nurse	Joint staff: Clinical & researcher	Public
P14	Male	Emergency Physician	Researcher	Public
P15	Male	Emergency Physician	Joint staff: Clinical & Academic	Public
P16	Female	Nurse	Joint staff: Clinical & Academic	Private
P17	Female	Emergency Physician	Academic	Public
P18	Male	Emergency Physician	Joint staff: Clinical & Academic	Public
P19	Female	Emergency Physician	Researcher	Public
P20	Female	None	Primary health care site facilitator	Public
P21	Female	None	Community representative	Public
P22	Female	Nurse	Community representative	Public
P23	Female	Public Health Physician	Academic leadership	Public
P24	Female	Physician	Academic leadership	Public
P25	Female	Emergency Physician	Academic	Public
P26	Male	Physician	Postgraduate student	Public
P27	Female	Physician	Postgraduate student	Public
P28	Male	Physician	Postgraduate student	Public
P29	Female	Out-of hospital provider	Postgraduate student	Public
P30	Male	Out-of hospital provider	Postgraduate student	Public

After the 30th interview, data redundancy was considered reached, with no new insights emerging. Analysis of the full dataset produced four overarching categories that captured key perspectives on the barriers and enablers of engaged research within the Western Cape EC community. These categories represent interconnected relational, structural, and normative factors that shape how engaged research is understood and practised in this setting, rather than discrete or opposing constructs. These categories were: (1) *Cultivating relationships and spaces for engagement*; (2) *Overstretched and under-resourced*; (3) *Systems*,* structures*,* and scholarship*; and (4) *The rules of engagement*. These categories were derived through systematic coding and abstraction, with an extract of the coding tree provided in Table 2 to illustrate the progression from codes to sub-categories and overarching categories within the analytical process.

**Table 2:** Extract of the coding tree illustrating the analytical process

Meaning unit	Condensed meaning unit	Code	Sub-category	Category
“I guess there needs to be, in so far as there needs to be investment in the relationship with the Community, there certainly has to be an investment in the relationship between those on the academic platform and those on the service platform.”	There needs to be investment in the relationship with the community and between the academic and service platforms	Community relationships required	Investment in relationships	Cultivating relationships and spaces for engagement
“I also think that we don’t know how to do it. If you have to say to me, even though I said it looks like I have. experience with engaged research I don’t know how to do it. I don’t know how to start. I’m just following myself blindly and thinking wow, how can I be this engaged scholar”	I don’t know how to do engaged research or how to start, even though I seem experienced	Limited engagement expertise	Learning How to Engage	Overstretched and under-resourced
“Different institutions all have different applications. Some of them have manual applications, some of them you just sent the documents, some of them have a specific online portal”	Different institutions have different application processes—manual, email, or online portal	Differing institute approvals	Bureaucracy as a Barrier	Systems, structures, and scholarship
“We haven’t set enough ground rules internally as to how it should happen. And I think we’ve all had our fingers burnt at this stage”	We haven’t set internal ground rules, and we’ve all been burnt by it	Documented rules of engagement needed	Setting the rules of engagement	The rules of engagement

### Cultivating relationships and spaces for engagement

#### Investment in relationships

Participants frequently emphasised that engaged research relies on developing strong, intentional relationships grounded in mutual respect and ongoing dialogue. This was viewed as particularly important between academics and those delivering services, but participants also stressed that these relationships should extend to the wider community, including patients, to support meaningful engagement.

There needs to be investment in the relationship with the Community…a more deliberate discussion between the clinicians and the academics so that there is alignment – P17; Academic

You also need to find out what are the dynamics within the community. What are the challenges? Who is the community that you’re going to work with? – P21; Community representative

The idea was raised that organisational structures may outline who is involved in research, yet this does not always translate into real relationships. Participants noted that meaningful engagement depends on direct connection, which is often missing.

Without strong relationships and the communication they enable, awareness of ongoing activities within the Western Cape EC community remains limited. Participants noted that valuable research and service initiatives often go unnoticed, reducing opportunities for collaboration, participation, and wider impact.

…there’s a lot more that we can do to highlight the work that is being done….I think there’s a lot of good work that sometimes gets done, but is not highlighted and people are not aware of it – P23; Academic leadership

I mean if you go and ask the consultant emergency physicians within [Hospital X] and you asked them what research the others are doing and they don’t, it’s not widely shared. – P8; Researcher

Participants consistently stressed that communication and relationships are fundamental to engaged research. Without them, interactions may remain guarded, with stakeholders reluctant to share openly or invest in collaboration. A lack of trust can also restrict access to facilities, resources, and opportunities for meaningful involvement.

It’s relationship and it’s conversation. You cannot, in my view, report to be an engaged scholar or a knowledge producer, if you are not fostering relationships with the communities – P1; Researcher

…a lot of it came down to personal relationships and an existing relationship….if you don’t know one another…then there’s always this kind of elephant in the room. Are you going to say something that we don’t like? or are you actually gonna sideline us and put out an article that says we’re rubbish? – P8; Researcher

### Creating place and space for discussion

Participants highlighted the need for regular spaces where stakeholders could meet, talk openly, and work through shared priorities. Such forums were viewed as essential for relationship building, bridging communication gaps, and strengthening engagement. These interactions did not need to be highly structured, but should allow for accessible, face-to-face conversation and the organic development of ideas.

The biggest problem is not really having a space or place to discuss the problems and help shape those into research questions because a lot of my experience has been that researchers use a very different language to frontline clinicians and managers and we don’t always really gel…. So I think it’s about creating an ongoing space where people have the time to actually engage. – P2; Researcher

For some, particularly those working closely with patients and community groups, in-person interaction was essential where digital access was limited. These settings were also likened to personal relationships, where informal conversation helps build trust and sustain connections over time.

In person. In person. In person….There isn’t any other way for community. Most of our communities are not on e-mail. They do not have a computer. They do not have a laptop. – P21; Community representative

…if you think about a partnership that you have with your best friend, sometimes you have coffee and you talk about other people. Sometimes you have coffee and you talk about your own problems and solutions that you need to work on. And sometimes you talk just to touch base to see what is happening… – P1; Researcher

Consistency was also emphasised, with participants cautioning that infrequent or irregular meetings risk being deprioritised and losing momentum.

… if we can have this regular conversation, it can facilitate many of the other things that we are worried about like the capacity building, relationship building, mutual trust and so on. – P1; Researcher

So the research day obviously is a great forum, but it’s once a year, there’s not much engagement with the researchers available because it’s quite a tight program – P28; Postgraduate student

#### Inclusive research dissemination for broader impact

Participants emphasised the need to broaden dissemination beyond traditional academic forums, which are often only attended by researchers rather than those who use or are affected by the work. While conferences and formal meetings remain useful, they frequently exclude frontline workers, service users, and other community stakeholders. Participants therefore called for more accessible, inclusive gatherings that support open dialogue and shared learning. One suggestion was a Western Cape EC “research imbizo,” drawing on the isiZulu verb *biza*, meaning to summon or call, referring to a community meeting where people come together to discuss shared issues.

we see that when we have research meetings and conferences to disseminate, the room is filled with other researchers. Not really the end users. – P14; Researcher

…a starting point is to have maybe a Western Cape research imbizo thing where everybody just sort of comes together who are interested in doing research – P16; Clinical & academic

### Overstretched and under-resourced

#### Competing priorities, limited time & the cost of engagement

Time and financial pressures were closely linked and widely described as major obstacles to engaged research. Heavy clinical and academic workloads with little protected time, pushes research into after-hours work and limits the continuity and relationship-building that engaged approaches require. Limited funding compounds this problem, with researchers frequently needing to self-fund activities, take unpaid leave, or avoid engagement-focused projects altogether. Without sufficient resources and time, the more complex, relationship-driven work central to engaged research becomes difficult to initiate and sustain.

we don’t have time, you know, I’m paid by the province, when must I do my research? Is it done between 2:00 and 3:00 in the morning. – P24; Academic leadership

…we don’t actually have the tools in terms of researchers and in terms of financial backing and monetary resources….They are probably very expensive research questions to answer. – P3; Clinical & academic

#### Learning how to engage

Another challenge was the misconception that engaged research is intuitive, when it in fact requires specific skills and experience. Some were unsure how to begin or felt expected to engage without adequate preparation. Many highlighted the need for formal teaching, mentorship, and guidance from experienced facilitators to build the capacity of both new and established researchers.

Theres this notion that within medicine, for example see one, do one and teach one…So its like “Oh yeah Dr X you are now a joint staff member, yippee, but without actually capacitating you to know exactly what it is that is expected of you. – P24; Academic leadership

I also think that we don’t know how to do it…. I don’t know how to start. I’m just following myself blindly and thinking wow, how can I be this engaged scholar – P1; Researcher

Participants also highlighted that such capacity-building should extend beyond researchers to patients and community representatives, emphasising that training can help community members better understand research processes, their potential benefits, and how to participate meaningfully.

the training has helped us to understand what is the research, what research entails and what is the benefits for community…first we didn’t understand what would the benefits be. Would it benefit us? – P21; Community representative

### Systems, structures, and scholarship

#### Bureaucracy as a barrier

Approval processes were frequently described as slow and burdensome, with differing requirements across institutions, facilities, and sectors. These inconsistencies, together with long timelines, were seen as particularly damaging for engaged research, which depends on timely collaboration and ongoing relationships. In several cases, delays discouraged stakeholders from working together or led researchers to abandon engagement at certain sites. Participants also noted that approval at senior levels does not always translate into practical access to facilities, data, or staff. When mechanisms to operationalise approvals are unclear, researchers encounter further barriers that disrupt engagement efforts and create additional frustration for both researchers and communities.

One of the huge constraints to research is the research processes in the Western Cape government. It is tardy. It is long, it’s just not an efficient structure… You decide to drop a particular facility because it’s taken so long to get approval. – P24; Academic leadership

Different institutions all have different applications. Some of them have manual applications, some of them you just sent the documents, some of them have a specific online portal – P13; Clinical & researcher

…if we have approval from the CEO of a hospital to run our research study, getting access to the facility is not a given…. the CEO says yes, you can do research in that facility, but then there’s no process or opportunity afforded for you to even get a badge to access the facility for your staff or access to the medical records systems… – P14; Researcher

#### Degree research as divider and driver

It was commonly reported that research in the Western Cape EC community is largely driven by postgraduate degree requirements. While this maintains steady activity, this was observed to place supervisors in a reactive position, following student-led priorities rather than pursuing coordinated agendas. The short timelines and qualification-driven nature of degree projects were also seen as limiting, as candidates often choose achievable or simpler topics over longer-term, engagement-focused work.

They [students] will do something that is a dream that they’ve always had…it’s very difficult sometimes swaying people from that pathway – P3; Clinical & academic

so many students pick a topic just because it’s an easy topic to get through so they can get the qualification…a project that is simplistic as can be for the sake of ticking the boxes. – P11; Governance

However many emphasised that degree research can still foster engagement, as many postgraduates live and work within the Western Cape EC system and are closely connected to local realities.

….there are many opportunities for engaged scholarship in the sense that our PhD students and Masters students are people living and working in the Western Cape system – P1; Researcher

Stakeholders noted that degree projects have often lacked strategic direction, with studies emerging in isolation. To address this, participants proposed linking smaller projects to broader, ongoing engagement initiatives, with senior researchers providing continuity to sustain relationships and ensure community priorities remain central.

we’ve not had a strategic intent and students arrive and they think of a topic and we supervise that topic…that creates a disconnect on what is thought to be important and what is being studied – P6; Researcher

In order for us to really commit to meaningful engaged research, it’s just my feeling that perhaps the MMeds need to fit in as part of a broader research initiative or bigger project – P25; Academic

I can plug in students as they come through on whatever the problem is. And I’m the person with a relationship and the engagement and the co-creation part might not take place when the students there but the student could still do studies – P6; Researcher

Some acknowledged that a more structured approach to aligning degree projects might reduce opportunities for idea generation and independent learning. However, this risk was generally viewed as minor compared to the benefits of producing meaningful, relevant work, and many, including students, felt that clearer direction would be welcomed.

being given a topic is not as empowering as being given the space to appreciate your thoughts, processes as well…. if you are too prescriptive, it is going to take away the opportunity for research literacy – P27; Postgraduate student

the only thing I would say that was lost out on was like the concept of idea generation. But that’s so tiny in comparison to what I learned, because my research actually was meaningful. As opposed to doing something really small and silly just to pass. – P29; Postgraduate student

### The rules of engagement

#### Learning from historical disconnects

Participants noted that research in the Western Cape EC community has not always prioritised engagement, with past examples of local and international researchers working in isolation and involving communities only at the point of dissemination. In some instances, communities felt exploited when external researchers collected data without consultation or ongoing involvement, reinforcing the need for more accountable and collaborative approaches.

historically the model of researchers, it’s quite singular in terms of you or your team just thinking about the problem…the only time we really engage is when we go to present our results at the end, you know, in a conference or a meeting – P14; Researcher

I could think of a few examples where foreign researchers, probably with good intentions, came in and kind of grabbed the data and did what they wanted without properly consulting and used the local communities and then kind of scuttled off back to their home country and churned out publications – P8; Researcher

#### Acknowledging the value of community contributions

Community members stressed that part of improving engagement involves fair compensation, emphasising that they are owed recognition and tangible benefit for their contributions. This was widely accepted as both justified and an important step towards building trust and stronger research relationships. The need to demonstrate the real-world value of engaged research was also raised. Showing visible impact was felt to build motivation, reduce apprehension, and foster a stronger sense of shared purpose across the community.

If a researcher is coming into my community and they are taking my time or needing me to have some expenses made, they need to cover the cost. – P20; Site facilitator

There must be something in the budget for communities, so that communities know that we are appreciated….you don’t just come to communities and you use our information. I think those were some reasons for the barriers or fences that we put up – P21; Community representative

Folks could have an opportunity to understand what we’re doing, see it in action. Some folks who were initially apprehensive to provide informed consent, later saw how actually valuable it was… to say look as a consequence of your participation here’s what we’ve discovered that is now helpful to our community at large – P14; Researcher

#### Setting the rules of engagement

Participants emphasised the need for clear rules and expectations for all parties involved in engagement projects. Without this, past experiences had left people feeling let down, with a lack of transparency and shared understanding undermining trust and limiting collaboration.

We haven’t set enough ground rules internally as to how it should happen. And I think we’ve all had our fingers burnt at this stage – P8; Researcher

It was widely agreed that engagement should begin with a process of negotiation to establish how collaboration will take place. This includes agreeing on the frequency and format of interactions and setting clear terms of reference to define the rules of engagement in a way that is acceptable to all parties involved.

the community will guide you as to how it should look. Is it a monthly meeting? You know, is it a quarterly meeting. Would they just want to be annually made aware of the research process? So I think in the beginning a lot of it would have communication with the community – P20; Site facilitator

I think the needs to be terms of reference when it gets established. So therein you would be able to give the rules of engagement and the purpose …. – P1; Researcher

## Discussion

Our study aimed to explored stakeholders’ perspectives on the barriers to and enablers of engaged research within the Western Cape EC community. Overall, engagement was viewed as both necessary and achievable, but dependent on sustained relationships, clear expectations, supportive institutional structures, and shared accountability.

Strengthening engaged research requires attention to several interconnected factors, beginning with the central role of relationships in shaping collaboration. Cultivating relationships emerged across all stakeholder groups as essential to meaningful and sustainable engagement. This reflects the wider literature showing that effective collaboration relies on trust built through ongoing, respectful interaction [40–42]. Relationship building is not a once-off activity but a continuous process throughout the research cycle, fostering rapport, mutual understanding, and enduring connections [40–42]. It is unsurprising that stakeholders from the Western Cape emphasised this, given that the Ukuphepha Transformational Model of Community Engagement, developed in the province and widely used across local academic institutions, serves as a guiding framework for socially responsive research [43]. The model places community readiness and relationship building at its foundation, reflecting what Le Dantec and Fox [44] describe as “the work that occurs before the work”: immersing in communities, demonstrating commitment, and addressing personal and institutional barriers to genuine collaboration.

Participants’ reflections that meaningful relationships with some community members remain limited highlight a gap between engagement principles and their real-world application. Similar challenges have been documented elsewhere, where patients and frontline clinicians are often marginalised in research processes and describe feeling out of touch with research [45–47]. Such findings, consistent with participants’ perspectives, reinforce calls for more inclusive and reciprocal engagement, ensuring that stakeholders see the relevance and benefit of research. This requires active facilitation through actions such as demonstrating tangible outcomes, widening dissemination channels, and promoting shared learning and participation [45, 46].

The role of degree research in engaged research is contentious. Participants noted its potential to support engagement through high output and regular project cycles, particularly when aligned with community priorities. Yet, as the literature recognises, it can also become one-sided, with academic goals overshadowing community needs. Without careful planning, even well-intentioned projects risk causing more harm than benefit [47, 48]. In response to such challenges, institutions have developed structured university–community partnership initiatives to support more equitable collaboration. One such initiative is the Knowledge Co-op, established at the University of Cape Town within our study region [49]. Modelled on the European Science Shop concept, it provides a platform for communities to propose their own questions, which are then available as potential research projects that may be taken up, though student participation remains voluntary. Some participants in our study not only suggested building on such initiatives but advanced this further, proposing that incoming degree research within departments associated with EC should by default align with pre-existing, engaged projects. Although such an approach may be unpopular with some students preferring increased research independence, as reflected by our participants, the broader view may be that it is a worthwhile trade-off to ensure research remains meaningful and responsive to community needs.

Stakeholders in our study highlighted the need for protected time and better alignment of engagement activities with existing clinical workflows, recommendations which are echoed in previous studies examining stakeholder participation [45, 50]. Participants described the benefit of having structured opportunities to learn how to participate in research. This reflects suggestions in the literature for dedicated “How to get involved” sessions, designed to explain practical pathways into engagement [46]. Building on this, the concept of implementing a continuum of engagement for clinicians has been suggested, allowing individuals to select levels of involvement according to their capacity and interest. Frameworks such as that adapted by Goldstein et al. [50] delineates engagement from minimal to participatory roles with indicative time commitments, alongside examples of expected responsibilities and tasks. Such frameworks offer a practical approach to integrating research engagement within routine professional responsibilities.

Delays and frustrations with ethical and governance approvals were identified as significant barriers to engaged research. Such challenges are amplified in engaged studies, which are often multi-site and collaborative, requiring approval across several institutions and sectors. Variations in application formats and review criteria can substantially delay progress and discourage collaboration [51, 52]. Research on multi-site studies shows that more than 75% of application time may be spent on administrative duplication, including reformatting documents, repeating ethics forms, and managing site-specific approvals, without improving study design or participant safety [51]. Larger collaborative projects also face higher costs, with one study reporting that increasing the number of participating centres by less than fivefold resulted in an almost twentyfold rise in administrative costs relative to the overall study budget [51]. These structural inefficiencies are compounded by a methodological mismatch. Although engaged research shares the same ethical objectives as institutional review boards, its iterative and adaptive nature does not align easily with approval systems designed for linear, pre-specified protocols. The need to adapt methods in response to community input often necessitates amendments and extended timelines. Echoing longstanding calls for reform [53] our stakeholders questioned whether fragmented review processes meaningfully enhance protection, and highlighted the importance of centralised review mechanisms, standardised templates, and reviewer training in engagement principles [52, 53]. Streamlining these processes is essential to ensure that ethical oversight protects participants without unintentionally obstructing the very collaboration and inclusivity that engaged research seeks to achieve.

The expectation that researchers should naturally know how to conduct engaged research, despite limited opportunities to develop the necessary skills, was viewed as a major barrier to meaningful collaboration. The lack of formal training further reinforces this gap. The wider literature similarly highlights the need for targeted education and mentorship as a crucial step toward strengthening research partnerships [45, 54, 55]. Notably, stakeholders in our study highlighted that this training should not just be limited to academic stakeholders. Participants emphasised the value of capacity building for non-academic partners, patients and community leaders, so they can engage confidently and equitably in research processes. This dual-focus approach, providing skills and knowledge to both researchers and community members, can be seen as key to balancing power dynamics and fostering genuine mutual understanding between all partners.

Participants emphasised that balancing power between stakeholders within research partnerships is fundamental to meaningful engagement. Historical experiences of non-engaged research have shown how communities can be harmed when treated as subjects rather than partners, leading to long-standing feelings of exploitation that, as raised by community members in our study, unfortunately persist today [52]. A clear message from participants was that rebuilding confidence in engagement requires transparency and mutual accountability; each party must understand their role, responsibilities, the process of collaboration, and what they can expect in return. The literature reinforces this call for clearly defined and jointly agreed foundations for collaboration, established before the start of any project. These should specify who is involved, expected contributions, anticipated benefits including fair compensation, and shared responsibility for disseminating results [45, 50, 56]. While the details may evolve as relationships mature, it is adherence to these rules of engagement that will ultimately enable trust to develop and meaningful partnerships to endure.

This study’s findings should be viewed within its limitations. As a qualitative project, these findings reflect the perspectives of those who chose to participate and the positionality of the research team. Participants already engaged in research may be overrepresented, and the team’s involvement in the Western Cape EC could have shaped both responses and interpretation. Reflexive measures, including maintaining an audit trail and regular team discussions, were used to examine assumptions and minimise potential bias. The use of online interviews may have influenced the depth of interaction and conducting interviews exclusively in English may have limited participation or nuanced expression for individuals more fluent in isiXhosa or Afrikaans. However, English is widely used as the primary language of professional communication within the EC and academic communities in the province. These factors should be considered when interpreting the findings.

## Conclusion

Meaningful engagement in research depends on sustained relationships, shared accountability, and clearly defined rules of engagement that balance power between academic and non-academic partners. This is especially critical in regions where disparities are pronounced, where trust must form the foundation for genuine collaboration. Achieving this requires time, resources, and institutional commitment to reform approval systems, align workflows, and build capacity across all participants. Embedding these principles throughout the research cycle, from design to dissemination, offers a path toward equitable, enduring partnerships that strengthen both knowledge generation and community impact within EC.

## Supplementary Information

Below is the link to the electronic supplementary material.

Supplementary Material 1

## Data Availability

Data in the form of anonymised interview transcripts supporting this study’s findings are available from the corresponding author on reasonable request.

## Abbreviations

EC: Emergency Care
HIC: High-income countries
LMIC: Low- and middle-income country
PPI: Patient and public involvement
