# Supplementary material for: Setting the rules of engagement: a qualitative study of challenges and enablers of engaged research in a South African emergency care system
Source: Res Involv Engagem. 2026 Apr 15;12:67. doi: 10.1186/s40900-026-00885-6 (PMC13192058; doi:10.1186/s40900-026-00885-6)
Supplement: Supplementary file 1 — Supplementary Material 1 [file 40900_2026_885_MOESM1_ESM.docx]

## Discussion Schedule

**Exploring the barriers and facilitators to engaged research within the Western Cape Emergency Care Community: A qualitative study**

Notes to Interviewer:

Instructions to the interviewer are displayed in *italics* and questions to be read to the participant are displayed in **bold.** Prompts, if required, are contained within the textboxes following each question.

Facilitation:

*Before beginning each interview refamiliarise yourself with this document and the interview process. The primary researcher will conduct and lead each interview, guiding the discussion, taking notes where appropriate and operating all audio recording software or equipment. The purpose of each interview is to obtain original unbiased opinions until it is agreed saturation has been reached.*

Preparation:

*For online interviews – Ensure a stable internet connection is established for both parties and all online software and recording equipment is tested prior to commencing the interview.*

*For in-person interviews – Ensure a quiet, private area is selected, offer participants refreshments and check that they are as comfortable as possible prior to commencing the interview.*

*Both – Ensure that all consent forms have been filled and that the participant has agreed to participate as well as audio recording of the interview.*

Confidentiality statement:

**After starting the recording I will begin with a brief confidentiality statement just to cover what is mentioned in the consent form and then well begin the interview is that ok. And as part of that for the purpose of the interview you will be participating P10....**(*start recording)* **The purpose of this interview is to gain insight into your experiences with engaged research and your views on the factors influencing its use. Therefore, there are no right or wrong responses to the questions asked, please just provide your personal views. Prior to the interview, you were provided with a full brief of the study as well as a consent form. Please remember you are welcome to stop the interview and withdraw your consent to participate at any point. We will not record your name in post-interview transcription and will perform an anonymised analysis. So for the purposes of the recording, this interview is being conducted with Participant x. Ok is that all clear.** *Allow time for any questions on confidentiality.*

Session introduction:

*Start the interview by briefly introducing yourself, and the purpose of the interview and allow the participant to introduce themselves, however, reiterate that they should not state his/her name). You may start the session as follows:*

**Perfect well thank you for agreeing to be interviewed today and taking the time to discuss the topic of engaged research. We will begin with a brief introduction and background before proceeding with the main questions of the interview. If at any point a question is unclear or you would like clarification or further explanation, please don’t hesitate to ask.**

**To start you could state your participant number and your gender for demographic purposes and then introduce yourself by providing a brief background on your role within the WC EC community.**

| Prompts: |
| --- |
| - Demographics: Age and gender - Qualification(s) - Years of experience in current position - Number of research projects involved in +/- published - What type of research have you been involved in before? - *Ensure the participant provides insight into whether they consider themselves a mainly knowledge/research producer or mainly knowledge/research user* |

**Can provide details of your previous experience in research generally and whether you would consider yourself a research user or producer. – Research experience**

**So as mentioned the purpose of our interview today is to discuss the topic of engaged research. Engaged research generally considered to be the application of academic work and expertise through engagement with non-academic constituents of a specific community to co-create knowledge for mutual benefit.**

**The value of engaged scholarship is increasingly recognised but there is very little information on the perceptions and practical application of engaged research and the factors impacting its success. The aim of our study is to explore the current practice of engaged research within the Western Cape Emergency Care (WC EC) community and identify barriers and facilitators to its implementation.**

**So to start can I ask if you have any prior knowledge or experience with engaged research and if so what your though thought are about the concept of engaged research?**

| Prompts: |
| --- |
| - Is it a scholarship you encounter regularly - Have you ever been directly involved in an engaged research project - If so, tell me about your experience - What does the term engaged research mean to you? - What do you think the importance of engaged research is? |

**What do you believe are the most important factors that could facilitate engaged research?**

| Prompts: |
| --- |
| - What characteristics are important in the parties involved? - What do you think is important in establishing trust? - What preparation and agreements are important prior to projects - What stages are important during the lifespan of a project - Other examples:   Organisational support, advisory boards, capacity for interaction, training, funding |

**What do you believe are the prohibiting barriers currently in place preventing the use and success of engaged research?**

| Prompts: |
| --- |
| - What negative characteristic of researcher producers and/or users prevents engagement - What do you think is lacking in the preparation phase currently - What do you think is lacking during engaged research projects - Other examples:   Organisation support, advisory boards, capacity for interaction, training, funding |

**What are some practical solutions you think could be implemented to increase and improve the level of engaged research conducted?**

| Prompts: |
| --- |
| - Prompts based on previous answers |

**Outside of academics and frontline emergency care clinicians, is there any person or role that you think will be important to interview or other areas in order to help us explore more deeply the concept of engaged research in emergency care?**

Conclusion of Interview: *Once it is apparent no further new information will be obtained the interview may be concluded with the following:*

**Thank you once again for agreeing to participate in today’s interview. The information and insight you have provided is and will be of great value to our overall project. Please do not hesitate to contact the research team if you wish to clarify anything regarding the interview or the progress of the project.**
